# Disordered Microbial Communities in Asthmatic Airways

**DOI:** 10.1371/journal.pone.0008578

**Published:** 2010-01-05

**Authors:** Markus Hilty, Conor Burke, Helder Pedro, Paul Cardenas, Andy Bush, Cara Bossley, Jane Davies, Aaron Ervine, Len Poulter, Lior Pachter, Miriam F. Moffatt, William O. C. Cookson

**Affiliations:** 1 National Heart and Lung Institute, Imperial College London, London, England; 2 Department of Respiratory Medicine, Connolly Hospital, Dublin, Ireland; 3 Instituto Gulbenkian de Ciência, Instituto de Tecnologia Química e Biológica, Oeiras, Portugal; 4 Department of Mathematics, University of California, Berkeley, California, United States of America; Institut de Pharmacologie et de Biologie Structurale, France

## Abstract

**Background:**

A rich microbial environment in infancy protects against asthma [1], [2] and infections precipitate asthma exacerbations [3]. We compared the airway microbiota at three levels in adult patients with asthma, the related condition of COPD, and controls. We also studied bronchial lavage from asthmatic children and controls.

**Principal Findings:**

We identified 5,054 *16S rRNA* bacterial sequences from 43 subjects, detecting >70% of species present. The bronchial tree was not sterile, and contained a mean of 2,000 bacterial genomes per cm^2^ surface sampled. Pathogenic Proteobacteria, particularly *Haemophilus* spp., were much more frequent in bronchi of adult asthmatics or patients with COPD than controls. We found similar highly significant increases in Proteobacteria in asthmatic children. Conversely, Bacteroidetes, particularly *Prevotella* spp., were more frequent in controls than adult or child asthmatics or COPD patients.

**Significance:**

The results show the bronchial tree to contain a characteristic microbiota, and suggest that this microbiota is disturbed in asthmatic airways.

## Introduction

Asthma is a heterogeneous syndrome of intermittent wheeze and airway inflammation that affects 300 million individuals worldwide. Although its causes are unknown, many studies suggest a role for microbiota in its aetiology [4]. Viral infections are important inducers of seasonal exacerbations of asthma [5], but there is circumstantial evidence that bacterial infections may also play a role. Asymptomatic neonates whose throats are colonized with *Streptococcus pneumoniae*, *Haemophilus influenzae*, or *Moraxella catarrhalis* are at increased risk for recurrent wheeze and asthma early in life [6]. These same bacteria have consistently been associated with exacerbations of both asthma [7] and chronic obstructive pulmonary disease (COPD) [8]. The response of asthmatics to antibiotics also suggests the importance of acute and chronic bacterial infections in the pathogenesis of disease [9]. Epidemiological research has consistently indicated that a rich microbial environment in early life confers protection against the development of asthma [1], suggesting the need to understand the extent and nature of normal airway flora.

Apart from asthma, respiratory infections, excluding tuberculosis, cause 6% of the global burden of disease and each year 4.2 million people die of lower respiratory infections. Significantly, death in the 1918–1919, 1957 and 1968 influenza pandemics resulted most commonly from secondary bacterial pneumonia caused by organisms presumed to be previously present in the respiratory tract [10], [11].

Chronic Obstructive Pulmonary Disease (COPD) shares many features with asthma and is the fourth leading cause of death worldwide. Infectious exacerbations of the disease are a frequent cause of death, and chronic infection causes a progressive decline of lung function.

Thus, despite strong evidence to implicate bacterial infections in the course and pathogenesis of airway diseases, it is unfortunate that a systematic study of organisms in the airways has been lacking [12].

Only 1% of all bacteria can be cultured in the laboratory [13], so culture is no longer the gold standard for the diagnosis of infections. Culture-independent molecular methods have already shown that the microbiota of humans is far greater in extent than previously recognised [14], [15], [16]. Humans are recognised to have evolved relationships with their symbiotic bacteria that are essential for health. Ecological changes altering this symbiosis can result in disease [17].

We therefore used molecular analysis of the polymorphic bacterial *16S-rRNA* gene to characterize the composition of bacterial communities from the airways of adult subjects including patients with asthma and COPD. We sought replication of findings from these adults in an additional study of children attending clinics for therapy-resistant asthma.

## Results

Twenty-four adult subjects were studied, including 5 patients with COPD, 11 patients with asthma and 8 control subjects with no previous history of asthma or COPD and an FEV1≥95% predicted (Table 1). Sterile dry cotton-headed swabs were used to sample from the nose and the oropharynx (OP) of all subjects (n = 24), and bronchoscopy via the nasal route was used to obtain duplicate cytology brushings within the left upper (LUL) in 23 subjects. All subjects were free of clinical infection at the time of the study.

**Table 1:** Adult Patient Characteristics.

	Asthma	COPD	Control
Number	11	5	8
Age (Mean+/−SD)	37.6+/−18.2	57.0+/−12.3	52.9+/−11.1
Sex (% male)	72.7%	60.0%	37.5%
Current smoking (%)	9.1%	80.0%	25.0%
Pack Years (Mean+/−SD)	0.6+/−1.4	41.0+/−28.8	9.0+/−17.4
FEV1 % Predicted (Mean+/−SD)	81.0+/−21.6	51.2+/−8.0	100.8+/−8.6
% Reversibility (Mean+/−SD)	9.3+/−11.6	9.8+/−4.8	3.0+/−4.7
DLCO (Mean+/SD)	91.2+/−6.2	60.6+/−13.1	88.0+/−12.1
Medication (%)			
ICS/LABA	100.0%	60.0%	0.0%
Tiotropium	9.1%	40.0%	0.0%
SABA	90.9%	60.0%	0.0%
Theophylline	18.2%	0.0%	0.0%
Oral steroids	9.1%	0.0%	0.0%
Singulair	27.3%	20.0%	0.0%
Severity (%)*			
Severe	27.3%		
Intermittent	27.3%		
Mild/Moderate	45.5%		

* based on the GINA criteria (http://www.ginasthma.com/).

We sequenced clone libraries from the *16S rRNA* PCR products for 23, 24 and 21 samples from the nose, OP and LUL respectively. Very weak amplification products were obtained from three subjects with the lowest bacterial copy numbers and we did not identify bacterial sequences from these libraries. After excluding possible chimeras, 1,081 nasal, 1,100 OP and 1068 LUL sequences were analysed further.

Distinct individual bacterial sequences (defined as operational taxonomic units (OTUs)) were identified with the DOTUR program. We estimated bacterial diversity, or the number of different types of bacteria (OTUs) likely to be present, with Chao 1 statistics (using a cut-off of ≥97% sequence identity). This analysis suggested the presence of 126 OTUs (95% Confidence Interval [CI] 97.7–194.8) in the nasal samples, 229 (95%CI 142.9–434.1) OTUs in the OP and 128 (95%CI 106.9–179.0) in the LUL samples. These estimates indicated that we had identified 62% of all OTUs in the nose and 68% of LUL OTUs, but only 35% of the diversity in the OP. The values in the nose and LUL are comparable with studies estimated to have identified 68.3% and 74.0% of OTUs in the human distal oesophagus [18] and forearm superficial skin [19] respectively.

**Figure 1:**
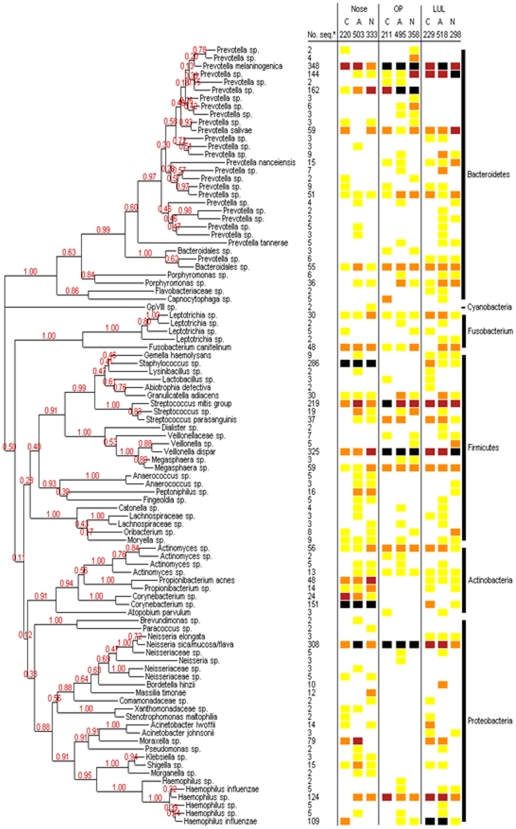
Phylogenetic analysis of bacterial *16S rRNA* DNA from the nose, oropharynx (OP) and left upper lobe (LUL) in adult patients with COPD (C) or asthma (A) and healthy controls (N). The numbers of *16S rRNA* gene phylotypes (OTUs) were calculated at 97% sequence identity and single-sequence OTUs omitted. OTU designations are located at the termination of each branch and represent potential organism names. Abundance of OTUs in each subject is indicated by different coloured squares (Yellow = 1 single OTU, Orange = 3–10%, Red = 10–20% and Black≥20%).

We combined nose, OP and LUL sequences together to produce a global DOTUR analysis for the airways. This identified 190 OTUs of which 97 had more than a single sequence. Highly abundant OTUs belonged to the genera *Prevotella*, *Streptococcus*, *Staphylococcus*, *Neisseria*, *Corynebacterium* and to *Haemophilus spp.* (Figure 1).

**Figure 2:**
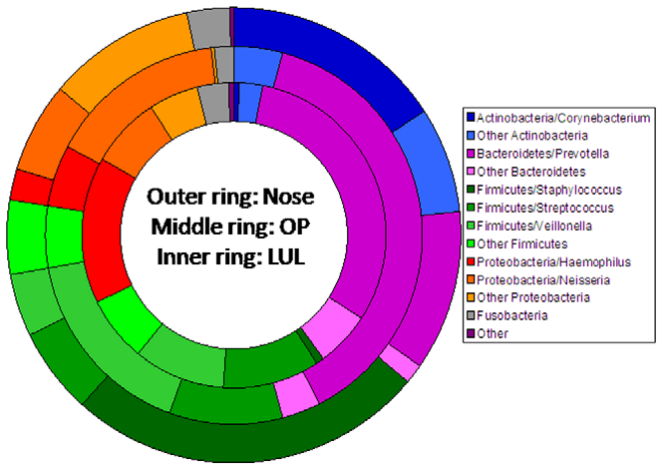
Percentage distribution of common phyla and genera at different airway levels (nose, OP and LUL), subdivided into the seven most frequent genera (*Croynebacterium*, *Prevotella*, *Staphylococcus*, *Streptococcus*, *Veilonella*, *Haemophilus and Neisseria*) found in the samples.

We used cladistic analysis to examine the distribution of phyla and highly abundant genera at different airway levels (Figure 2 and Table 2). Nasal specimens were highly significantly characterised by *Actinobacteria* (particularly *Corynebacterium* spp.) (*P_c_*<10^−51^) and *Firmicutes* (particularly *Staphylococcus* spp.) (*P_c_*<10^−89^). The most common bacteria in the OP were *Bacteroidetes* (particularly *Prevotella* spp.) (*P_c_*<10^−25^), and LUL samples contained many more *Haemophilus* spp. than the other sites (*P_c_*<10^−10^).

**Table 2:** Cladistic association analysis: *P*-values for differences in phyla and genera between airway levels in adult subjects.

PHYLA	LUL	OP	NOSE	*P* * LUL vs. OP	*P* LUL vs. nose	*P* OP vs. Nose
Proteobacteria	302	230	207		2.08E−03	
Bacteroidetes	392	459	138		1.82E−35	1.98E−50
Firmicutes	297	347	447		1.44E−07	7.57E−03
Fusobacteria	38	18	34			
Actinobacteria	34	45	253		4.23E−44	6.30E−39
**GENERA**						
Actinobacteria/Corynebacterium	6	0	171		2.36E−42	1.53E−52
Other Actinobacteria	28	45	82		4.25E−04	
Bacteroidetes/Prevotella	330	423	121		2.36E−26	1.93E−47
Other Bacteroidetes	62	36	17		3.91E−04	
Firmicutes/Staphylococcus	9	0	277		4.38E−74	1.46E−90
Firmicutes/Streptococcus	110	106	65			
Firmicutes/Veillonella	104	184	49	6.02E−03	1.03E−02	1.04E−17
Other Firmicutes	74	57	56			
Proteobacteria/Haemophilus	167	60	24	1.39E−11	9.30E−27	2.69E−01
Proteobacteria/Neisseria	79	167	69	3.17E−05	1.28E+03	7.39E−08
Other Proteobacteria	56	3	114	5.59E−11	1.51E−02	1.15E−28

The numbers of sequences are shown for each split level.

* Only significant P values are shown. The significance levels have been Bonferroni corrected for multiple comparisons.

**Figure 3:**
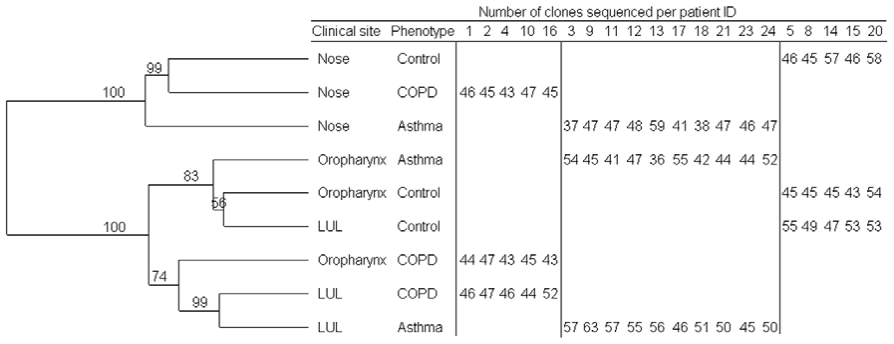
Bacterial community comparisons from the nose (N), oropharynx (OP) and left upper lobe (LUL) in adult patients with COPD or asthma and healthy controls.

A whole microbial community comparison showed the nasal microbiota for all phenotypes to be clearly distinct from that of the OP and LUL (Figure 3). The OP of healthy controls clustered with OP samples of asthmatics and also with the controls' LUL. The LUL samples of patients with asthma and COPD clustered together, along with the OP samples of patients with COPD. These findings suggested that the microbiota of the healthy LUL was similar to that of the healthy OP, but the LUL microbiota differed from normal OP and LUL samples in the presence of asthma and COPD. The analysis also suggested that the microbiota in patients with COPD was altered in the OP as well as the LUL, perhaps due to exposure to cigarette smoke both regions.

We next studied the LUL samples in more detail for their relationship to airway disease. Both series of 23 specimens from the left upper lobe (LUL) were analysed in triplicate for bacterial counts. The *16S rRNA* copy number estimates indicated a range of 2–7,500 bacterial genomes per 40ng total DNA, or a mean of 2.2×10^3^ bacterial genomes per cm^2^ surface sampled (range 62–2.1×10^5^) based on an estimated brushed area of 2 cm^2^ and one copy of the 16S rRNA gene per genome. The repeat LUL brushings gave highly reproducible counts (Spearman's rho = 0.667, *P*<0.001). We excluded sample-specific PCR inhibition in subjects with low copy numbers by demonstrating amplification of human DNA with equivalent intensity for all samples.

Our mean estimate of 2,000 bacterial genomes per cm^2^ surface sampled from LUL brushings is comparable with estimates from the upper two thirds of the small intestine [20]. The airway counts are an order of magnitude below the 50,000 bacteria/cm^2^ identified by *16S rRNA* amplification of scrapings from normal skin [16], although the maximum count of 20,000 we observed in an asthmatic subject approximates this figure.

The genome copy number estimates were significantly lower in smokers (Mann-Whitney U test Z = −3.92, *P*<0.001). Similar effects of smoking were observed in the duplicate samples (Z = −4.25, *P*<0.001) and in the nasal counts (Z = −2.38, *P* = 0.016). The specimens from the RLL were in the same range as for the LUL. No differences in the counts were found with respect to age or sex. It may be relevant that cigarette smoking has also been shown to influence bacterial growth in inflammatory bowel disease (IBD) [21].

**Figure 4:**
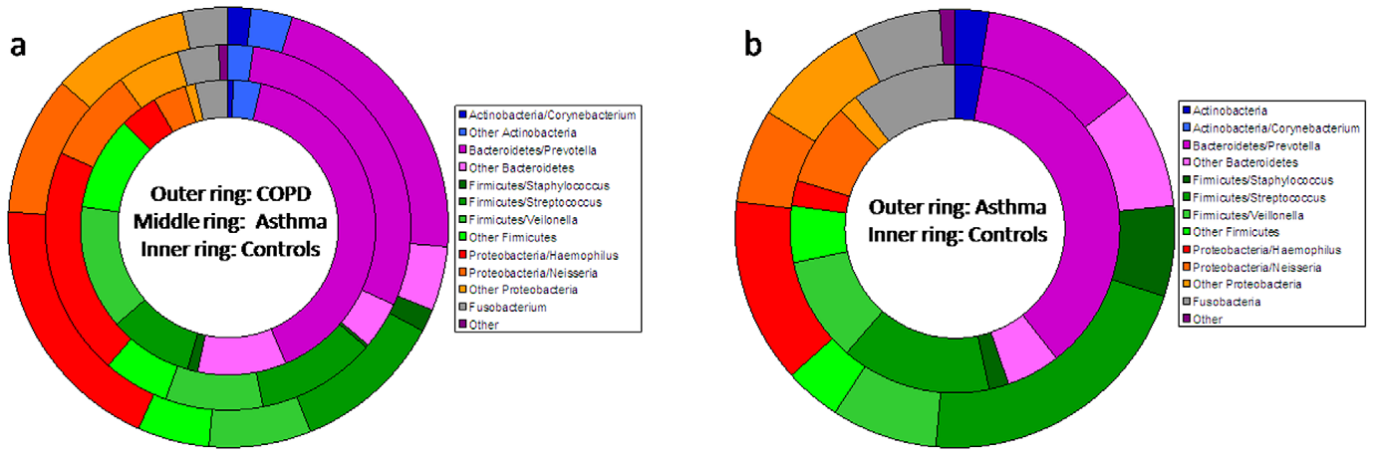
Distribution of common phyla and genera in diseased and normal bronchi. a) Distribution of the phyla from sheathed bronchoscopic brushings of the LUL for patients with asthma and COPD and normal subjects, subdivided into the seven most frequent genera (*Croynebacterium*, *Prevotella*, *Staphylococcus*, *Streptococcus*, *Veilonella*, *Haemophilus and Neisseria*). b) Distribution of the phyla from bronhco-alveolar lavage (BAL) in children with difficult asthma and controls.

We performed cladistic association analysis to test for differences in the frequency of particular phyla and genera between subject groups (Figure 4a and Tables 3 and 5). We found members of the phylum Proteobacteria to occur highly significantly more often in the COPD (*P_c_*<10^−14^) and asthma (*P_c_*<10^−13^) groups compared to controls (Table 3). In contrast, Bacteroidetes members were more common in controls compared to diseased subjects (*P_c_*<10^−4^ controls versus COPD; *P_c_*<10^−2^ controls versus asthma). Members of the genus *Haemophilus* were most strongly associated with the presence of COPD (*P_c_*<10^−4^) and asthma (*P_c_*<10^−7^) (Table 1d).

**Table 3:** Cladistic association analysis: *P*-values for differences in phyla and genera from left upper lobe brushings between adult subject groups.

PHYLA	COPD	Asthma	Control	*P* * COPD vs. Controls	*P* Asthma vs. Controls
Proteobacteria	94	181	27	7.70E−15	2.16E−14
Bacterioidetes	62	179	151	3.38E−05	7.17E−03
Firmicutes	60	134	103		
Fusobacteria	8	19	11		
Actinobacteria	11	12	11		
**GENERA**					
Actinobacteria/Corynebacterium	4	0	2		
Other Actinobacteria	7	12	9		
Bacteroidetes/Prevotella	51	158	121	7.55E−03	
Other Bacteroidetes	11	21	30		
Firmicutes/Staphylococcus	4	2	3		
Firmicutes/Streptococcus	26	56	28		
Firmicutes/Veillonella	18	45	41		
Other Firmicutes	12	31	31		
Proteobacteria/Haemophilus	46	108	13	2.06E−05	1.17E−08
Proteobacteria/Neisseria	24	44	11		
Other Proteobacteria	24	29	3	9.22E−04	
Fusobacterium	8	19	11		

The numbers of sequences are shown for each split level.

* Only significant P values are shown. The significance levels have been Bonferroni corrected for multiple comparisons.

In order to confirm if the same bacteria were relevant in health and disease in a different set of patients we studied an additional 13 (5 female) asthmatic and 7 (3 female) control individuals recruited through a tertiary referral clinic for difficult asthma at the Royal Brompton Hospital (Table 4). The median age of the asthmatic children was 11.8±2.8 years and that of the controls was 11.3±5.7. None of the subjects had been taking antibiotics prior to examination and all subjects were considered free of infection. Broncho-alveolar lavage (BAL) was performed through a fibre-optic bronchoscope, DNA extracted, and clone libraries created and sequenced.

**Table 4:** Characteristics of childhood subjects.

ID	Status	Age	Sex	Ethnicity	FEV1%	Therapy
1	Asthma	9	f	A	44	budes 1200/m5/form36/
2	Asthma	8	m	Mix	79	Seretide 250 2p bd/pred 15 eod m5
3	Asthma	11	m	C	98	symb 200 2P bd/theo300bd/Azithromycin 250 3×wk/pred 6.25
4	Asthma	11	f	C	83	salb prn/becotide 50 2PBD
5	Asthma	15	m	C	86	Seretide 250 2PBD/
6	Asthma	9	m	C	51	seretide 250 2p bd/m5/hydro 12.5 tds
7	Asthma	14	f	C	65	Seretide 250 2PBD/pred 10 eod/
8	Asthma	11	m	C	88	Servent 25 2pbd/flix 250 2pbd/PRED 15 EOD
9	Asthma	12	f	A	73	Seretide 1252pbd/calcich 500bd/m5/pred 10od
10	Asthma	11	m	Mix	63	symb 400 2 pbd/Slophyllin 125 bd/m5/pred 10 EOD
11	Asthma	15	m	A	N/A	pulm 200 6P BD/M4/serevent 25 3PBD/omep 10 BD/domp 3 TDs
14	Asthma	14	f	C	68	symb 400 2BB/M10/Slophyllin 250bd/domp 10tds/ome 20 od/sodium valp
15	Asthma	7	m	C	92	symb 200 5 pbd/m8/domp10 tds/omep20bd/azathiop75od
13	Stridor on exercise	13	m	C	84	Nil
52	laryngo and tracheomalacia	7	m	C	Normal	Nil
78	VSD, bronched during cardiac catheter	1	m	C	Normal	Nil
89	Recurrent haemoptysis, no resp symptoms	17	f	C	Normal	Nil
98	Oesophageal reflux, productive cough	11	m	C	78	Ventolin prn/domp/omepr/lansoprazole
300	Replacment of pacemaker	17	f	C	Normal	Nil
348	Stridor, prev. surgery for vascular ring	12	f	C	Normal	Nil

We obtained 1135 and 670 sequences respectively for the asthmatic and control individuals. Based on 97% sequence identity, this resulted in 97 (124.3 95%CI 108.3–163.2) and 68 (85.1 95%CI 73.7–119.4) OTUs for the asthma and control individuals. Taking all subjects together, 131 OTUs were detected.

We found from the distribution of the phyla that Proteobacteria were more frequent in asthmatic children compared to controls (*P_c_*<10^−13^), whereas Bacterioidetes were more common in the controls than in asthmatics (*P_c_*<10^−16^) (Figure 4b and Table 5). At the level of genera, the asthmatic subjects were characterised by excess of *Haemophilus* spp. (*P_c_*<10^−12^) and *Staphylococcus* spp. (*P_c_*<10^−2^), whereas *Prevotella* spp. were more frequent in controls (*P_c_*<10^−30^).

**Table 5:** Cladistic association analysis: *P*-values for differences in phyla and genera between broncho-alveolar lavage in difficult childhood asthmatics and control subjects.

PHYLA	Control	Asthma	*P* * Asthma vs. Controls
Proteobacteria	83	331	6.45E−14
Bacterioidetes	282	239	1.13E−17
Firmicutes	218	454	
Fusobacteria	69	70	
Actinobacteria	18	27	
**GENERA**			
Bacteroidetes/Prevotella	247	137	3.23E−31
Other Bacteroidetes	35	102	
Firmicutes/Staphylococcus	13	74	7.16E−03
Firmicutes/Streptococcus	98	242	
Firmicutes/Veillonella	69	90	
Other Firmicutes	38	48	
Proteobacteria/Haemophilus	17	154	1.44E−13
Proteobacteria/Neisseria	53	77	
Other Proteobacteria	13	100	1.01E−06
Fusobacteria	69	70	

The numbers of sequences are shown for each split level.

* Only significant P values are shown. The significance levels have been Bonferroni corrected for multiple comparisons.

## Discussion

Our results challenge the traditional medical teaching that the lower airways are sterile, and suggest that bronchial tree contains a characteristic microbial flora that differs between health and disease. The concept of sterility comes from an age with limited experimental access to healthy airways and was based on 120 year old culture techniques which are now recognised to detect a small percentage of the bacteria present in complex samples [13]. Indeed, the major colonists in our control subjects were anaerobes such as *Prevotella spp.*, which can only be grown with difficulty in culture. Microbiota are ubiquitous even in the most hostile environments, and it would be extraordinary if the lower airway able to maintain sterility in the presence of high volume airflow through a damp open communication with the oropharynx.

Our results show strong similarities in the airway microbiota between adult and paediatric asthmatics and controls (Figure 4), despite sampling by two alternative methods (bronchial brushing and BAL). Strikingly, members of the phylum Proteobacteria were very strongly associated with airway disease in both groups. The phylum contained the important potential pathogens *Haemophilus*, *Moraxella* and *Neisseria* spp.

These associations, although robust, do not yet establish causality between the presence of pathogens and airways disease. The numbers of subjects are small and may not be representative of most cases of asthma. Our adult controls were healthy, but the childhood controls had clinical indications for their bronchoscopy including tracheomalacia, gastro-oesophageal reflux, and symptomatic VSDs which might all cause airway mucosal abnormalities. It is also not known to what extent past antibiotic usage in response to symptoms of airway disease may have determined the patterns of bacterial species we have detected. It is also possible that the presence of pathogens in asthmatic airways is secondary to mucosal abnormalities of as yet unknown cause.

Despite these cautions, it is striking that *Haemophilus*, *Moraxella* and *Neisseria* spp. found by us in asthmatic airways have also been shown to carry increased risk for asthma in early life when found in neonatal throat swabs [6]. It should therefore be considered possible that the presence of these pathogens may have some influence on chronic airway inflammation, even when below the threshold for routine culture. In addition, the presence of a low copy-number yet dominant population of potential pathogens may provide a substrate for full blown bacterial infection following otherwise self-limiting viral infections [22] as well as following pandemic influenza [10], [11].

We are not aware of any trials of antibiotics in asthma other than in the management of acute exacerbations [9], but It may be relevant that protracted course of antibiotics are required for successful therapy of the asthma overlap syndrome of chronic wet cough [23]. The availability of effective vaccines against *Haemophilus influenzae* might also open new therapeutic possibilities for individuals whose airways disease is characterised by the continued presence of this pathogen.

Our data suggest that *Staphylococcus* spp. are also present in excess in the airways of children with difficult asthma, and it is likely that wider exploration of children and adults with asthma will identify a range of organisms associated with the disease, including *Mycoplasma* spp. and *Chlamydia* spp. [24] and *Streptococcus* spp. [6], and possibly defining subtypes of the syndrome. Longitudinal studies are desirable to follow changes in the microbiota during the acute exacerbations that are a prominent feature of asthma and COPD.

Bacteroidetes (particularly *Prevotella* spp.) were more common in control subjects than in diseased individuals. *Prevotella* are Gram-negative anaerobes recognised to be part of the normal oral [25] and vaginal flora and are the predominant anaerobic Gram-negative bacilli isolated from respiratory tract infections and their complications. They have been found in high numbers by *16S-rRNA* sequencing in the lungs of children with cystic fibrosis [26]. Our results suggest that *Prevotella* and *Veillonella* spp. are a distinctive component of the normal flora of the lung as well as other moist epithelia, and that their presence in normal airways may not have been generally recognised because of their requirement for anaerobic culture.

The reduction of Bacteroidetes in patients with asthma and COPD compared to controls may also be relevant to disease. An investigation of patients with IBD showed a 10-fold decrease in total bacterial load and a diminished quantity of Bacteroidetes compared with controls [21], consistent with findings in our patients. It may be relevant that *Prevotella* spp. directly inhibit the growth of other bacteria [27]. Commensal bacteria elicit tonic signals in the gut epithelium that prevent activation of innate and adaptive immune responses [28], [29], and commensal bacteria down-regulate immune responses to pathogens in the nasal mucosa [30]. It is possible that similar mechanisms may characterise healthy airways.

## Materials and Methods

Approval for the adult study was given by the Connolly Hospital Ethics Committee, and full informed consent was obtained from each patient in writing. Approval for the study of the pediatric patients was given Royal Brompton Hospital Ethics Committee, and written informed consent from parents and age-appropriate assent from children was obtained in each case.

Twenty-four adult subjects were studied, consisting of 5 patients with COPD, 11 patients with asthma and 8 control subjects with no previous history of asthma or COPD and an FEV1≥95% predicted (Table 1). Asthma severity was based on the GINA criteria (http://www.ginasthma.com/). Smoking was defined as current/recent (within the past two years), past (non-smoker for more than two years) and never. Subjects were included in the study regardless of smoking status. All patients were of European descent and from Dublin. The patients received their usual medical treatment in the 24 hours prior to this investigation. No patients were taking antibiotics at the time of the study, and all subjects were considered to be free of clinical infections.

Sterile dry cotton-headed swabs were used to sample from the nose and the oropharynx (OP) of all subjects (n = 24). Following Propofol as a short-acting sedative agent and local Lidocaine anaesthesia to the upper airway, bronchoscopy was performed via the nasal route. Olympus Endotherapy disposable sheathed cytology brushes (Model number BC-202D-5010) 5 mm diameter and 10 mm length were used to brush within the left upper (LUL) and right lower lobes (RLL). Duplicate brushings were done for the LUL, attempting in each case to reach a more distal area. One patient with severe asthma was unable to tolerate a bronchoscopy. LUL brushings were therefore performed for 23 subjects. Fourteen of the subjects (five controls; three COPD subjects and six asthma subjects) were sufficiently comfortable to allow sampling of their RLLs. Approximately 2 cm^2^ of bronchial mucosa was covered by each brushing. Samples were either processed immediately or stored in 2 ml tubes over night at −20°C prior to being processed.

We also studied thirteen paediatric patients with difficult asthma and seven non-asthmatic controls (Table 2). Difficult asthma was defined as symptoms requiring a rescue bronchodilator on at least three days per week, despite treatment with at least 1600 µg/day of inhaled budesonide (or equivalent), as well as regular long acting β2 agonists (or a previous unsuccessful trial of long acting β2 agonists), and/or regular prednisolone [31]. None of the subjects were taking antibiotics at the time of the study and all were considered to be free of clinical infections. Fifteen children were Caucasian, three were of African origin and one child was of mixed origin (Table 4). The study had full ethical approval and all subjects or their parents gave written informed consent.

Bronchoscopies in the paediatric subjects were performed as previously described [31]. For the BAL, three aliquots of normal saline (1 ml/kg, maximum 40 ml) were instilled in the right middle lobe through the bronchoscope and the fluid retrieved by mechanical suction. Between 0.4–8ml of BAL fluids were retrieved and centrifuged and the pellet kept for subsequent DNA extraction. In the case of three patients the pellets had been used for a different study. We therefore again centrifuged the remaining supernatants and processed these secondary pellets.

Samples were suspended in 450µl lysis solution (20 mg/ml lysozyme; 20 mM Tris·HCl, pH 8.0; 2 mM EDTA) and incubated for 1h at 37°C with occasional vortexing. Subsequent DNA extraction was performed as instructed by the manufacturer (DNeasy, Qiagen). The purified DNA was resuspended in 40µl of analar water and the DNA concentration determined using a Nanodrop ND-1000 spectrophotometer. For long term storage samples were kept at −20°C. Working stocks containing 20 ng/µl and 10 g/µl total DNA were prepared for samples from the LUL, RLL and nose and these stocks were stored at 4°C.

To quantify the bacterial DNA content of each sample, 2µl of each prepared working stock was used as template in PCR reactions containing *16S rRNA* gene primers 63F-5′ GCAGGCCTAACACATGCAAGTC-3′ and 355R-5′ CTGCTGCCTCCCGTAGGAGT-3′ (final primer concentration 0.2 µM) with reaction and cycling conditions as previously described [16]. A standard curve was included, amplifying serial dilutions of known quantities of *Escherichia coli* cells in the presence of 20 ng/µl human DNA. For the left upper and right lower lobe samples the functions used to calculate copy numbers were: C_t1_ = −3.62x+35.46 with R^2^ = 0.994; C_t2_ = −3.76x+36.30 with R^2^ = 0.996 and C_t3_ = −3.73x+36.05 with R^2^ = 0.996. For the nasal swabs, the function used was: C_tN_ = −3.70x+35.91 with R^2^ = 0.997. Copy numbers were calculated with *E. coli* having 4,500,000 bp. Initial experiments revealed no inhibition of amplification of *E. coli* in the presence of human DNA and confirms findings described elsewhere [16]. Differences in genome count numbers were assessed using non-parametric tests in SPPS 16.0 for Windows.

For the sequencing analysis *16S rRNA* genes were amplified using the primers 339F-5′-ACTCCTACGGGAGGCAGCAGT-3′ [15] and 907R-5′-CCGTCAATTCMTTTGAGTTT-3′ [32]. 25µl PCR reactions were set up containing 2µl of template, 2.5µl 10× buffer (Roche Applied Biosystems), and final concentrations of 1.5 mM Mg, 6% DMSO, 0.5 µM primers, 0.8 mM total dNTP (0.2 mM each) and 1U HotStart Polymerase (Roche Applied Biosystems). Cycling conditions were one cycle of 95°C for 5 min, then 32 cycles of 95°C for 30s, 55°C for 30s and 72°C for 60s followed by a final extension of 72°C for 5 min.

Following amplification, the PCR products were separated on a 1% agarose gel, and bands corresponding to the target product (∼550bp) were excised. Cloning and sequencing of the PCR products was possible for 21, 23, 24 and 20 primary LUL, nose, OP and BAL samples respectively. A low concentration of amplification product of 3 samples was attributed to low bacterial counts and prevented reliable sequencing. PCR products were extracted from the gel slices using the Qiaquick extraction kit according to the manufacturer's instructions (Qiagen) and resuspended in 40µl of analar water and stored at 4°C until further use.

Purified PCR products were cloned into the pGEM-T Easy Vector (Promega) as per the manufacturer's instructions. Between 48 and 72 colonies per ligation were picked at random. Plasmid DNA was purified using the Qiagen Plasmid Miniprep Kit according to the manufacturer's instructions and sequenced using the primer SP6. In instances when sequence from the SP6 primer was of low quality, the alternate DNA strand was sequenced using the T7 primer. DNA sequence chromatograms were uploaded to the ribosomal database (http://rdp.cme.msu.edu/), vector sequences trimmed (using the LUCY program) and remaining sequences quality control checked (using PHRED). Sequences of high quality were downloaded, primer regions additionally trimmed and only full length sequences ranging from 359 up to 906 (*E. coli* numbering of the *16S rRNA* gene) were included for further analysis.

Sequences were aligned with NAST [33] and ClustalW. Alignments were manually curated and results checked for chimeras with Bellerophon Version 3 [34] on the Greengenes website (http://greengenes.lbl.gov). Using slightly adjusted standard settings (match length to core set = 400 and window size of 200) putative chimeras were identified and excluded from subsequent analyses.

Distance matrices calculating the pair wise distances of the high quality sequences was created on the green genes website (http://greengenes.lbl.gov/cgi-bin/nph-index.cgi), masking the hyper-variable regions with the lane mask utility. OTUs were designated using DOTUR (Distance based OTU and richness determination) [35] by the furthest-neighbour algorithm. Chao 1 richness measures of diversity were estimated by the same program. For the creation of the phylogenetic tree, a cut off of 97% was set and each OTU was then assigned an organism name based on the phylogenetic placement using sequence match of the ribosomal database (20 best match sequences define taxanomic rank) and compared to NCBI BLAST results for the percentage of sequence identity.

Cladistic association analysis was performed as a method for association mapping [36]. The approach was based on the observation that every edge in a phylogenetic tree defines a split of the OTUs. *P*-values were calculated for association between the groups and the split and for association between disease status of individuals and the split. Two-tailed Fisher's exact tests were performed for each split, followed by the application of a conservative Bonferroni correction [37] for multiple testing.

The UNIFRAC metric measures the distance between communities as the percentage of branch length that leads to descendants from only one of a pair of environments represented in a single phylogenetic tree, i.e. the fraction of evolution that is unique to one of the microbial communities. The metric thus reflects differences between the lineages that are adapted to live specifically in one environment or the others [38]. A phylogenetic tree based on *16S rRNA* DNA sequences was used as input to measure the difference between bacterial communities in asthma, COPD and controls by using UNIFRAC.

For Figure 1, the numbers of *16S rRNA* gene phylotypes (OTUs) were calculated at 99% sequence identity with the furthest neighbour clustering in the program DOTUR. Using a single sequence for each OTU, evolutionary distances were calculated with the Jukes-Cantor (tree-Markov) model and phlyogenetic trees determined by the Neighbour-Joining method, with 1000 trees generated. Bootstrap confidence levels were shown at the tree nodes for values for ≥50%. Designations of *Streptococcus mitis* and *Neisseria mucosa/sicca/flava* were as described previously [39], [40].

Bacterial community comparisons of PCR libraries were determined with weighted UniFrac and the relationships are displayed by the Unweighted Pair Group Method with Arithmetic mean (UPGMA). Sequences of individuals with the same phenotype and clinical site were pooled and sequence numbers annotated. The tree with 100% identity was used as input. Only individuals from whom all 3 sites resulted in bacterial sequences are included (20 of 24 individuals; 2,871 of 3,259 sequences).

Sequences are stored as GenBank accession GQ360090-GQ365143.
